# {(*E*)-4-Hy­droxy-*N*′-[phen­yl(pyridin-2-yl-κ*N*)methyl­idene]benzohydrazide-κ^2^ *N*′,*O*}bis­(nitrato-κ^2^ *O*,*O*′)copper(II)

**DOI:** 10.1107/S1600536811055772

**Published:** 2012-01-11

**Authors:** Rahman Bikas, Farhad Sattari, Behrouz Notash

**Affiliations:** a Young Researchers Club, Tabriz Branch, Islamic Azad University, Tabriz, Iran; b School of Physics, Iran University of Science and Technology, 16844 Tehran, Iran; c Department of Chemistry, Shahid Beheshti University, G. C., Evin, Tehran 1983963113, Iran

## Abstract

In the title compound, [Cu(NO_3_)_2_(C_19_H_15_N_3_O_2_)], the coordination geometry around the Cu^II^ ion can be described as distorted square-pyramidal, with two N atoms and one O atom from an (*E*)-4-hy­droxy-*N*′-[phen­yl(pyridin-2-yl)methyl­ene]benzohydrazide ligand and one nitrate O atom in the basal plane and one nitrate O atom at the apical site. The other two nitrate O atoms also bind to the Cu atom with long Cu—O distances [2.607 (4) and 2.853 (5) Å]. The crystal packing is stabilized by inter­molecular N—H⋯O and O—H⋯O hydrogen bonds.

## Related literature

For background to aroylhydrazones, see: Craliz *et al.* (1955 ▶). For pharmacological and catalytic applications of aroylhydrazones, see: Hosseini Monfared *et al.* (2010 ▶). For related structures, see: Huo *et al.* (2004 ▶); Kong *et al.* (2009 ▶); Mohd Lair *et al.* (2010 ▶); Shit *et al.* (2009 ▶); Yin (2008 ▶). For van der Waals radii, see: Bondi (1964 ▶).
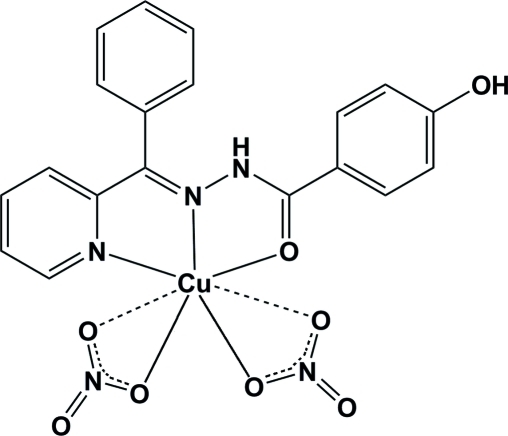


## Experimental

### 

#### Crystal data

[Cu(NO_3_)_2_(C_19_H_15_N_3_O_2_)]
*M* *_r_* = 504.91
Triclinic, 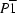

*a* = 9.881 (2) Å
*b* = 10.373 (2) Å
*c* = 11.964 (2) Å
α = 102.51 (3)°
β = 105.07 (3)°
γ = 111.16 (3)°
*V* = 1036.6 (6) Å^3^
*Z* = 2
Mo *K*α radiation
μ = 1.11 mm^−1^
*T* = 298 K
0.30 × 0.15 × 0.10 mm

#### Data collection

Stoe IPDS 2T diffractometer
Absorption correction: numerical (*X-SHAPE* and *X-RED32*; Stoe & Cie, 2005 ▶) *T*_min_ = 0.731, *T*_max_ = 0.897
11512 measured reflections
5533 independent reflections
4123 reflections with *I* > 2σ(*I*)
*R*_int_ = 0.099

#### Refinement

*R*[*F* ^2^ > 2σ(*F* ^2^)] = 0.052
*wR*(*F* ^2^) = 0.197
*S* = 1.13
5533 reflections
303 parameters
1 restraint
H atoms treated by a mixture of independent and constrained refinement
Δρ_max_ = 0.84 e Å^−3^
Δρ_min_ = −0.64 e Å^−3^

### 

Data collection: *X-AREA* (Stoe & Cie, 2005 ▶); cell refinement: *X-AREA*; data reduction: *X-AREA*; program(s) used to solve structure: *SHELXS97* (Sheldrick, 2008 ▶); program(s) used to refine structure: *SHELXL97* (Sheldrick, 2008 ▶); molecular graphics: *ORTEP-3* (Farrugia, 1997 ▶); software used to prepare material for publication: *WinGX* (Farrugia, 1999 ▶).

## Supplementary Material

Crystal structure: contains datablock(s) I, global. DOI: 10.1107/S1600536811055772/hy2498sup1.cif

Structure factors: contains datablock(s) I. DOI: 10.1107/S1600536811055772/hy2498Isup2.hkl

Additional supplementary materials:  crystallographic information; 3D view; checkCIF report

## Figures and Tables

**Table 1:** Hydrogen-bond geometry (Å, °)

*D*—H⋯*A*	*D*—H	H⋯*A*	*D*⋯*A*	*D*—H⋯*A*
N3—H3*A*⋯O5^i^	0.88 (4)	2.20 (5)	2.866 (6)	132 (4)
N3—H3*A*⋯O4^i^	0.88 (4)	2.31 (4)	3.180 (5)	171 (3)
O2—H2*A*⋯O8^ii^	0.82	1.95	2.766 (5)	174

Symmetry codes: (i) 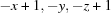; (ii) 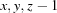.

**Fig. 1.**
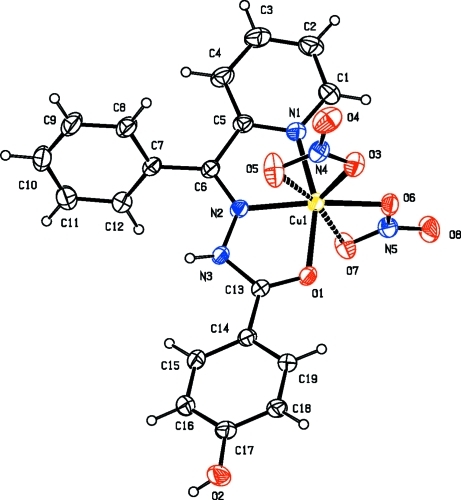
The molecular structure of the title compound. Displacement ellipsoids are drawn at the 30% probability level.

**Fig. 2.**
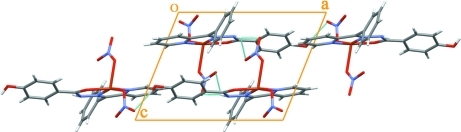
The packing diagram of the title compound showing hydrogen bonds as blue dashed lines.

## supplementary crystallographic information

### Comment

Hydrazone ligands, a class of Schiff-base compounds, derived from the condensation of acid hydrazides (*R*–CO–NH–NH_2_) with aromatic 2-pyridyl aldehydes or ketones are important tridentate O, N, N-donor ligands. The coordination chemistry and biochemistry of aroylhydrazones, *R*–CO–NH–N=CH–*R*', have attracted increasing interest due to their chelating ability and pharmacological applications (Craliz *et al.*, 1955; Huo *et al.*, 2004; Kong *et al.*, 2009; Mohd Lair *et al.*, 2010; Shit *et al.*, 2009; Yin, 2008). Hydrazone ligands create environments similar to biological systems by usually making coordination through O and N atoms. The coordination compounds of aroylhydrazones have been reported to act as enzyme inhibitors and are useful due to their pharmacological and catalytic applications (Hosseini Monfared *et al.*, 2010). As part of our studies on the synthesis and characterization of aroylhydrazone compounds, we report here the crystal structure of a new copper complex obtained by the reaction of Cu(NO_3_)_2_.3H_2_O with (E)-4-hydroxy-N'-[phenyl(pyridin-2-yl)methylene]benzohydrazide (H*L*) in methanol.

The coordination geometry around the Cu^II^ ion can be described as disotorted five-coordinated square-pyramidal (Fig. 1). The square plane is constructed by two N atoms and one O atom from the hydrazone ligand and O6 from a nitrate group. The apical position is occupied by O3 atom of another nitrate group. There are also two secondary bonding interactions between the Cu atom and O7 and O5 of two nitrate groups (dashed lines in Fig. 1). These Cu···O distances are 2.607 (4) and 2.853 (5) Å for O7 and O5, respectively. They are shorter than sum of van der Waals radii of oxygen and copper atoms (2.92 Å; Bondi, 1964). The crystal packing of the title compound is stabilized by intermolecular N—H···O and O—H···O hydrogen bonds (Fig. 2, Table 1).

### Experimental

The H*L* ligand was prepared by refluxing a mixture of 2-benzylpyridine and 4-hydroxybenzohydrazide with equivalent molar ratio in 20 ml methanol. The mixture was refluxed for 3 h. The solution was then evaporated on a steam bath to 5 ml and cooled to room temperature. The obtained solids were separated and filtered off, washed with 5 ml of cooled methanol and then dried in air.

For preparing the title compound, the appropriate H*L* ligand (1.0 mmol) was dissolved in methanol (20 ml), then Cu(NO_3_)_2_.3H_2_O (1.1 mmol) was added and the solution was refluxed for 4 h. After cooling, the resulting green solution was filtered and evaporated at room temperature. X-ray quality crystals of the title compound were obtained by slow solvent evaporation.

### Refinement

H atom of the N—H group was found in difference Fourier map and refined isotropically. H atom of the O—H group and aromatic C—H groups were positioned geometrically and refined as riding atoms, with C—H = 0.93 and O—H = 0.82 Å and with *U*_iso_(H) = 1.2*U*_eq_(C) and 1.5*U*_eq_(O).

### Crystal data

**Table:**

[Cu(NO_3_)_2_(C_19_H_15_N_3_O_2_)]	*Z* = 2
*M**_r_* = 504.91	*F*(000) = 514
Triclinic, *P*1	*D*_x_ = 1.618 Mg m^−^^3^
Hall symbol: -P 1	Mo *K*α radiation, λ = 0.71073 Å
*a* = 9.881 (2) Å	Cell parameters from 5533 reflections
*b* = 10.373 (2) Å	θ = 1.9–29.2°
*c* = 11.964 (2) Å	µ = 1.11 mm^−^^1^
α = 102.51 (3)°	*T* = 298 K
β = 105.07 (3)°	Needle, green
γ = 111.16 (3)°	0.30 × 0.15 × 0.10 mm
*V* = 1036.6 (6) Å^3^	

### Data collection

**Table:**

Stoe IPDS 2T diffractometer	5533 independent reflections
Radiation source: fine-focus sealed tube	4123 reflections with *I* > 2σ(*I*)
graphite	*R*_int_ = 0.099
Detector resolution: 0.15 mm pixels mm^-1^	θ_max_ = 29.2°, θ_min_ = 1.9°
rotation method scans	*h* = −13→13
Absorption correction: numerical (*X-SHAPE* and *X-RED32*; Stoe & Cie, 2005)	*k* = −13→14
*T*_min_ = 0.731, *T*_max_ = 0.897	*l* = −16→16
11512 measured reflections	

### Refinement

**Table:**

Refinement on *F*^2^	Primary atom site location: structure-invariant direct methods
Least-squares matrix: full	Secondary atom site location: difference Fourier map
*R*[*F*^2^ > 2σ(*F*^2^)] = 0.052	Hydrogen site location: inferred from neighbouring sites
*wR*(*F*^2^) = 0.197	H atoms treated by a mixture of independent and constrained refinement
*S* = 1.13	*w* = 1/[σ^2^(*F*_o_^2^) + (0.1213*P*)^2^] where *P* = (*F*_o_^2^ + 2*F*_c_^2^)/3
5533 reflections	(Δ/σ)_max_ < 0.001
303 parameters	Δρ_max_ = 0.84 e Å^−^^3^
1 restraint	Δρ_min_ = −0.64 e Å^−^^3^

### Special details

**Table:**

Geometry. All e.s.d.'s (except the e.s.d. in the dihedral angle between two l.s. planes) are estimated using the full covariance matrix. The cell e.s.d.'s are taken into account individually in the estimation of e.s.d.'s in distances, angles and torsion angles; correlations between e.s.d.'s in cell parameters are only used when they are defined by crystal symmetry. An approximate (isotropic) treatment of cell e.s.d.'s is used for estimating e.s.d.'s involving l.s. planes.
Refinement. Refinement of *F*^2^ against ALL reflections. The weighted *R*-factor *wR* and goodness of fit *S* are based on *F*^2^, conventional *R*-factors *R* are based on *F*, with *F* set to zero for negative *F*^2^. The threshold expression of *F*^2^ > σ(*F*^2^) is used only for calculating *R*-factors(gt) *etc*. and is not relevant to the choice of reflections for refinement. *R*-factors based on *F*^2^ are statistically about twice as large as those based on *F*, and *R*- factors based on ALL data will be even larger.

### Fractional atomic coordinates and isotropic or equivalent isotropic displacement parameters (Å^2^)

**Table:**

	*x*	*y*	*z*	*U*_iso_*/*U*_eq_	
Cu1	0.70675 (5)	−0.09738 (4)	0.74518 (4)	0.03884 (16)	
O1	0.7184 (4)	−0.2036 (3)	0.5904 (2)	0.0440 (6)	
O2	0.6844 (5)	−0.4026 (4)	0.0445 (3)	0.0650 (9)	
H2A	0.7540	−0.3520	0.0256	0.098*	
O3	0.4467 (4)	−0.2228 (3)	0.6994 (3)	0.0566 (7)	
O4	0.2388 (4)	−0.1955 (4)	0.6201 (4)	0.0750 (10)	
O5	0.4417 (5)	−0.0900 (5)	0.5848 (4)	0.0811 (12)	
O6	0.7259 (3)	−0.2248 (3)	0.8443 (3)	0.0461 (6)	
O7	0.9656 (4)	−0.1034 (4)	0.8623 (3)	0.0595 (8)	
O8	0.9127 (4)	−0.2510 (4)	0.9654 (3)	0.0644 (9)	
N1	0.7192 (4)	0.0659 (3)	0.8753 (3)	0.0409 (6)	
N2	0.7711 (3)	0.0601 (3)	0.6788 (2)	0.0356 (5)	
N3	0.7779 (4)	0.0173 (3)	0.5644 (3)	0.0400 (6)	
N4	0.3756 (4)	−0.1701 (3)	0.6356 (3)	0.0455 (7)	
N5	0.8729 (4)	−0.1919 (4)	0.8921 (3)	0.0430 (6)	
C1	0.6978 (5)	0.0614 (5)	0.9806 (4)	0.0528 (9)	
H1	0.6719	−0.0267	0.9963	0.063*	
C2	0.7131 (7)	0.1835 (6)	1.0667 (4)	0.0654 (12)	
H2	0.6984	0.1783	1.1396	0.078*	
C3	0.7503 (7)	0.3120 (6)	1.0427 (5)	0.0703 (14)	
H3	0.7574	0.3945	1.0982	0.084*	
C4	0.7777 (6)	0.3203 (5)	0.9357 (4)	0.0529 (9)	
H4	0.8072	0.4085	0.9202	0.063*	
C5	0.7601 (4)	0.1944 (4)	0.8529 (3)	0.0389 (7)	
C6	0.7855 (4)	0.1873 (4)	0.7353 (3)	0.0365 (6)	
C7	0.8239 (4)	0.3152 (3)	0.6936 (3)	0.0371 (6)	
C8	0.7251 (5)	0.3824 (4)	0.6785 (4)	0.0507 (9)	
H8	0.6321	0.3449	0.6917	0.061*	
C9	0.7662 (6)	0.5059 (5)	0.6437 (5)	0.0605 (11)	
H9	0.6987	0.5493	0.6313	0.073*	
C10	0.9046 (6)	0.5646 (5)	0.6275 (4)	0.0602 (11)	
H10	0.9319	0.6492	0.6066	0.072*	
C11	1.0036 (6)	0.4994 (5)	0.6419 (4)	0.0571 (10)	
H11	1.0977	0.5394	0.6308	0.068*	
C12	0.9617 (5)	0.3721 (4)	0.6734 (4)	0.0480 (8)	
H12	1.0267	0.3257	0.6809	0.058*	
C13	0.7418 (4)	−0.1287 (4)	0.5217 (3)	0.0378 (7)	
C14	0.7340 (4)	−0.1929 (4)	0.3982 (3)	0.0372 (6)	
C15	0.7989 (5)	−0.1090 (4)	0.3319 (4)	0.0463 (8)	
H15	0.8528	−0.0070	0.3681	0.056*	
C16	0.7840 (5)	−0.1755 (4)	0.2136 (4)	0.0464 (8)	
H16	0.8290	−0.1188	0.1708	0.056*	
C17	0.7007 (5)	−0.3293 (4)	0.1578 (3)	0.0449 (8)	
C18	0.6355 (5)	−0.4144 (4)	0.2234 (4)	0.0474 (8)	
H18	0.5798	−0.5162	0.1867	0.057*	
C19	0.6544 (4)	−0.3464 (4)	0.3427 (3)	0.0420 (7)	
H19	0.6136	−0.4032	0.3869	0.050*	
H3A	0.762 (5)	0.060 (4)	0.510 (3)	0.042 (11)*	

### Atomic displacement parameters (Å^2^)

**Table:**

	*U*^11^	*U*^22^	*U*^33^	*U*^12^	*U*^13^	*U*^23^
Cu1	0.0537 (3)	0.0329 (2)	0.0368 (2)	0.02255 (18)	0.01981 (19)	0.01504 (16)
O1	0.0690 (16)	0.0362 (11)	0.0377 (12)	0.0304 (12)	0.0234 (12)	0.0157 (10)
O2	0.089 (2)	0.0500 (16)	0.0504 (16)	0.0209 (16)	0.0403 (17)	0.0075 (13)
O3	0.0596 (17)	0.0522 (16)	0.0630 (18)	0.0276 (14)	0.0200 (14)	0.0273 (14)
O4	0.0497 (18)	0.068 (2)	0.096 (3)	0.0300 (16)	0.0149 (18)	0.015 (2)
O5	0.069 (2)	0.079 (2)	0.095 (3)	0.0210 (19)	0.022 (2)	0.058 (2)
O6	0.0507 (14)	0.0443 (13)	0.0531 (15)	0.0243 (11)	0.0212 (12)	0.0265 (12)
O7	0.0511 (16)	0.0687 (19)	0.0625 (19)	0.0213 (14)	0.0227 (14)	0.0366 (16)
O8	0.073 (2)	0.082 (2)	0.066 (2)	0.0469 (19)	0.0297 (17)	0.0495 (19)
N1	0.0471 (16)	0.0399 (14)	0.0382 (14)	0.0211 (12)	0.0179 (12)	0.0125 (12)
N2	0.0459 (15)	0.0335 (12)	0.0286 (12)	0.0212 (11)	0.0110 (11)	0.0098 (10)
N3	0.0608 (18)	0.0340 (13)	0.0350 (14)	0.0275 (13)	0.0211 (13)	0.0146 (11)
N4	0.0447 (16)	0.0369 (14)	0.0431 (16)	0.0158 (12)	0.0061 (13)	0.0085 (12)
N5	0.0487 (16)	0.0496 (16)	0.0352 (14)	0.0258 (14)	0.0135 (12)	0.0182 (13)
C1	0.065 (3)	0.059 (2)	0.043 (2)	0.030 (2)	0.0250 (19)	0.0219 (18)
C2	0.090 (3)	0.078 (3)	0.047 (2)	0.048 (3)	0.038 (2)	0.023 (2)
C3	0.105 (4)	0.061 (3)	0.055 (3)	0.046 (3)	0.039 (3)	0.009 (2)
C4	0.070 (3)	0.046 (2)	0.0421 (19)	0.0315 (19)	0.0173 (18)	0.0065 (15)
C5	0.0451 (17)	0.0410 (16)	0.0310 (15)	0.0231 (14)	0.0115 (13)	0.0093 (12)
C6	0.0421 (17)	0.0336 (14)	0.0368 (15)	0.0208 (13)	0.0139 (13)	0.0114 (12)
C7	0.0438 (17)	0.0306 (14)	0.0346 (15)	0.0192 (13)	0.0098 (13)	0.0079 (11)
C8	0.055 (2)	0.0444 (19)	0.064 (2)	0.0316 (17)	0.0240 (19)	0.0214 (18)
C9	0.082 (3)	0.049 (2)	0.067 (3)	0.043 (2)	0.026 (2)	0.026 (2)
C10	0.087 (3)	0.0409 (19)	0.053 (2)	0.026 (2)	0.025 (2)	0.0228 (17)
C11	0.061 (2)	0.053 (2)	0.054 (2)	0.0182 (19)	0.024 (2)	0.0221 (19)
C12	0.054 (2)	0.0459 (18)	0.052 (2)	0.0259 (16)	0.0228 (17)	0.0208 (16)
C13	0.0446 (17)	0.0338 (15)	0.0416 (17)	0.0217 (13)	0.0195 (14)	0.0132 (13)
C14	0.0446 (17)	0.0371 (15)	0.0349 (15)	0.0229 (13)	0.0159 (13)	0.0121 (12)
C15	0.064 (2)	0.0337 (15)	0.0471 (19)	0.0236 (15)	0.0256 (17)	0.0154 (14)
C16	0.060 (2)	0.0460 (18)	0.0426 (18)	0.0264 (17)	0.0242 (17)	0.0205 (15)
C17	0.053 (2)	0.0437 (18)	0.0387 (17)	0.0232 (16)	0.0193 (15)	0.0099 (14)
C18	0.055 (2)	0.0349 (16)	0.050 (2)	0.0165 (15)	0.0273 (17)	0.0083 (14)
C19	0.0496 (19)	0.0387 (16)	0.0449 (18)	0.0213 (15)	0.0246 (16)	0.0159 (14)

### Geometric parameters (Å, °)

**Table:**

Cu1—N2	1.944 (3)	C4—C5	1.379 (5)
Cu1—N1	1.978 (3)	C4—H4	0.9300
Cu1—O6	1.983 (3)	C5—C6	1.483 (5)
Cu1—O1	1.993 (2)	C6—C7	1.475 (4)
Cu1—O3	2.268 (3)	C7—C12	1.383 (5)
O1—C13	1.256 (4)	C7—C8	1.388 (5)
O2—C17	1.339 (5)	C8—C9	1.386 (6)
O2—H2A	0.8200	C8—H8	0.9300
O3—N4	1.247 (4)	C9—C10	1.367 (7)
O4—N4	1.233 (5)	C9—H9	0.9300
O5—N4	1.232 (5)	C10—C11	1.373 (7)
O6—N5	1.295 (4)	C10—H10	0.9300
O7—N5	1.230 (4)	C11—C12	1.399 (6)
O8—N5	1.237 (4)	C11—H11	0.9300
N1—C1	1.339 (5)	C12—H12	0.9300
N1—C5	1.354 (5)	C13—C14	1.454 (5)
N2—C6	1.282 (4)	C14—C19	1.400 (5)
N2—N3	1.373 (4)	C14—C15	1.400 (5)
N3—C13	1.364 (4)	C15—C16	1.377 (5)
N3—H3A	0.88 (4)	C15—H15	0.9300
C1—C2	1.380 (6)	C16—C17	1.405 (5)
C1—H1	0.9300	C16—H16	0.9300
C2—C3	1.365 (7)	C17—C18	1.401 (5)
C2—H2	0.9300	C18—C19	1.378 (5)
C3—C4	1.389 (6)	C18—H18	0.9300
C3—H3	0.9300	C19—H19	0.9300
			
N2—Cu1—N1	80.16 (12)	C4—C5—C6	124.1 (3)
N2—Cu1—O6	158.77 (13)	N2—C6—C7	126.2 (3)
N1—Cu1—O6	97.76 (12)	N2—C6—C5	111.8 (3)
N2—Cu1—O1	79.23 (11)	C7—C6—C5	121.9 (3)
N1—Cu1—O1	159.40 (12)	C12—C7—C8	119.5 (3)
O6—Cu1—O1	101.39 (11)	C12—C7—C6	120.1 (3)
N2—Cu1—O3	116.69 (12)	C8—C7—C6	120.4 (3)
N1—Cu1—O3	90.60 (13)	C9—C8—C7	119.5 (4)
O6—Cu1—O3	84.35 (11)	C9—C8—H8	120.3
O1—Cu1—O3	98.75 (13)	C7—C8—H8	120.3
C13—O1—Cu1	113.6 (2)	C10—C9—C8	120.9 (4)
C17—O2—H2A	109.5	C10—C9—H9	119.6
N4—O3—Cu1	109.2 (2)	C8—C9—H9	119.6
N5—O6—Cu1	107.5 (2)	C9—C10—C11	120.3 (4)
C1—N1—C5	119.4 (3)	C9—C10—H10	119.8
C1—N1—Cu1	126.8 (3)	C11—C10—H10	119.8
C5—N1—Cu1	113.7 (2)	C10—C11—C12	119.5 (4)
C6—N2—N3	125.3 (3)	C10—C11—H11	120.3
C6—N2—Cu1	119.5 (2)	C12—C11—H11	120.3
N3—N2—Cu1	114.7 (2)	C7—C12—C11	120.2 (4)
C13—N3—N2	112.3 (3)	C7—C12—H12	119.9
C13—N3—H3A	118 (3)	C11—C12—H12	119.9
N2—N3—H3A	124 (3)	O1—C13—N3	119.5 (3)
O5—N4—O4	117.7 (4)	O1—C13—C14	121.7 (3)
O5—N4—O3	120.0 (4)	N3—C13—C14	118.9 (3)
O4—N4—O3	122.3 (4)	C19—C14—C15	118.9 (3)
O7—N5—O8	123.4 (4)	C19—C14—C13	117.8 (3)
O7—N5—O6	118.4 (3)	C15—C14—C13	123.3 (3)
O8—N5—O6	118.2 (3)	C16—C15—C14	120.8 (3)
N1—C1—C2	122.0 (4)	C16—C15—H15	119.6
N1—C1—H1	119.0	C14—C15—H15	119.6
C2—C1—H1	119.0	C15—C16—C17	119.8 (3)
C3—C2—C1	118.6 (4)	C15—C16—H16	120.1
C3—C2—H2	120.7	C17—C16—H16	120.1
C1—C2—H2	120.7	O2—C17—C18	116.6 (3)
C2—C3—C4	120.3 (4)	O2—C17—C16	123.4 (3)
C2—C3—H3	119.8	C18—C17—C16	119.9 (3)
C4—C3—H3	119.8	C19—C18—C17	119.6 (3)
C5—C4—C3	118.3 (4)	C19—C18—H18	120.2
C5—C4—H4	120.8	C17—C18—H18	120.2
C3—C4—H4	120.8	C18—C19—C14	120.9 (3)
N1—C5—C4	121.3 (3)	C18—C19—H19	119.5
N1—C5—C6	114.6 (3)	C14—C19—H19	119.5
			
N2—Cu1—O1—C13	−7.4 (3)	Cu1—N1—C5—C6	0.7 (4)
N1—Cu1—O1—C13	−7.8 (5)	C3—C4—C5—N1	−0.9 (7)
O6—Cu1—O1—C13	−165.8 (3)	C3—C4—C5—C6	−180.0 (4)
O3—Cu1—O1—C13	108.2 (3)	N3—N2—C6—C7	−5.0 (6)
N2—Cu1—O3—N4	−0.9 (3)	Cu1—N2—C6—C7	−176.1 (3)
N1—Cu1—O3—N4	78.4 (3)	N3—N2—C6—C5	175.6 (3)
O6—Cu1—O3—N4	176.1 (3)	Cu1—N2—C6—C5	4.4 (4)
O1—Cu1—O3—N4	−83.2 (3)	N1—C5—C6—N2	−3.2 (5)
N2—Cu1—O6—N5	−9.2 (4)	C4—C5—C6—N2	176.0 (4)
N1—Cu1—O6—N5	−92.0 (2)	N1—C5—C6—C7	177.3 (3)
O1—Cu1—O6—N5	80.4 (2)	C4—C5—C6—C7	−3.5 (6)
O3—Cu1—O6—N5	178.2 (2)	N2—C6—C7—C12	−58.8 (5)
N2—Cu1—N1—C1	−176.0 (4)	C5—C6—C7—C12	120.6 (4)
O6—Cu1—N1—C1	−17.4 (4)	N2—C6—C7—C8	123.5 (4)
O1—Cu1—N1—C1	−175.6 (4)	C5—C6—C7—C8	−57.1 (5)
O3—Cu1—N1—C1	67.0 (4)	C12—C7—C8—C9	−0.1 (6)
N2—Cu1—N1—C5	1.2 (3)	C6—C7—C8—C9	177.6 (4)
O6—Cu1—N1—C5	159.8 (3)	C7—C8—C9—C10	−1.9 (7)
O1—Cu1—N1—C5	1.6 (5)	C8—C9—C10—C11	1.9 (7)
O3—Cu1—N1—C5	−115.9 (3)	C9—C10—C11—C12	0.0 (7)
N1—Cu1—N2—C6	−3.3 (3)	C8—C7—C12—C11	2.0 (6)
O6—Cu1—N2—C6	−89.4 (4)	C6—C7—C12—C11	−175.7 (4)
O1—Cu1—N2—C6	176.9 (3)	C10—C11—C12—C7	−2.0 (7)
O3—Cu1—N2—C6	82.4 (3)	Cu1—O1—C13—N3	9.0 (4)
N1—Cu1—N2—N3	−175.4 (3)	Cu1—O1—C13—C14	−172.0 (3)
O6—Cu1—N2—N3	98.5 (4)	N2—N3—C13—O1	−5.0 (5)
O1—Cu1—N2—N3	4.8 (2)	N2—N3—C13—C14	176.1 (3)
O3—Cu1—N2—N3	−89.7 (3)	O1—C13—C14—C19	19.3 (5)
C6—N2—N3—C13	−173.3 (3)	N3—C13—C14—C19	−161.8 (3)
Cu1—N2—N3—C13	−1.7 (4)	O1—C13—C14—C15	−162.1 (4)
Cu1—O3—N4—O5	16.3 (5)	N3—C13—C14—C15	16.8 (5)
Cu1—O3—N4—O4	−164.1 (3)	C19—C14—C15—C16	0.5 (6)
Cu1—O6—N5—O7	−5.3 (4)	C13—C14—C15—C16	−178.0 (4)
Cu1—O6—N5—O8	174.7 (3)	C14—C15—C16—C17	1.0 (6)
C5—N1—C1—C2	1.3 (6)	C15—C16—C17—O2	−178.2 (4)
Cu1—N1—C1—C2	178.4 (4)	C15—C16—C17—C18	−1.1 (6)
N1—C1—C2—C3	0.4 (8)	O2—C17—C18—C19	177.0 (4)
C1—C2—C3—C4	−2.4 (9)	C16—C17—C18—C19	−0.3 (6)
C2—C3—C4—C5	2.6 (8)	C17—C18—C19—C14	1.8 (6)
C1—N1—C5—C4	−1.1 (6)	C15—C14—C19—C18	−1.9 (6)
Cu1—N1—C5—C4	−178.5 (3)	C13—C14—C19—C18	176.7 (4)
C1—N1—C5—C6	178.1 (3)		

### Hydrogen-bond geometry (Å, °)

**Table:**

*D*—H···*A*	*D*—H	H···*A*	*D*···*A*	*D*—H···*A*
N3—H3A···O5^i^	0.88 (4)	2.20 (5)	2.866 (6)	132 (4)
N3—H3A···O4^i^	0.88 (4)	2.31 (4)	3.180 (5)	171 (3)
O2—H2A···O8^ii^	0.82	1.95	2.766 (5)	174

Symmetry codes: (i) −*x*+1, −*y*, −*z*+1; (ii) *x*, *y*, *z*−1.
